# Redox-Driven Magnetic Regulation in a Series of Couplers in Bridged Nitroxide Diradicals

**DOI:** 10.3390/molecules30030576

**Published:** 2025-01-27

**Authors:** Fengying Zhang, Meiwen Song, Cheng Luo, Teng Ma, Yali Zhao, Boqiong Li, Yuxiang Bu

**Affiliations:** 1 Department of Materials Science and Engineering, Jinzhong University, Jinzhong 030619, China; 18835256270@163.com (M.S.); 15085595812@163.com (C.L.); mateng1022@tyust.edu.cn (T.M.); yaliz12@163.com (Y.Z.); lbq@jzxy.edu.cn (B.L.); 2 School of Chemistry and Chemical Engineering, Shandong University, Jinan 250100, China

**Keywords:** redox induction, magnetic regulation, magnetic character, nitroxide diradical, DFT calculation

## Abstract

Redox-induced magnetic regulation in organic diradicals is distinctly attractive. In this work, taking nitroxide radicals as spin sources, we predict the magnetic properties of 9, 10-anthraquinone, 9, 10-phenaquone, 9, 10-diazanthracene and 9, 10-diazepine-bridged molecular diradical structures in which the couplers are prone to dihydrogenation reduction at positions 9 and 10. As evidenced at both the B3LYP and M06-2X levels of theory, the calculations confirm that the magnetic transitions between ferromagnetism and antiferromagnetism can take place for 9, 10-anthraquinone and 9, 10-diazanthracene-bridged diradicals after dihydrogenation. The differences in the magnetic behaviors and magnetic magnitudes of 9, 10-anthraquinone and 9, 10-diazanthracene-bridged diradicals before and after dihydrogenation could be attributed to their noticeably different spin-interacting pathways. As for 9, 10-phenaquone and 9, 10-diazepine-bridged diradicals, the calculated results indicate that the signs of their magnetic exchange coupling constants *J* do not change, but the magnitudes remarkably change after dihydrogenation. The connecting bond character and spin polarization are crucial in explaining the different magnetic magnitudes of these designed diradicals. In detail, shorter bonds and larger spin polarization are responsible for strong magnetic coupling. In addition, the diradical with an extensively π-conjugated structure can effectively promote magnetic coupling. The McConnell’s spin alternation rule is the key to understanding the observed ferromagnetism and antiferromagnetism of these diradicals. The work provides useful information for the rational design of redox-regulated magnetic molecular switches.

## 1. Introduction

Organic magnetic materials have attracted wide attention from experimental and theoretical chemists because of their unique properties [1,2,3,4,5,6,7,8,9,10,11,12]. Rajac et al. [4] prepared an organic diradical consisting of two Blatter radicals and found that this diradical possessed robust thermal stability and good electrical conductivity. Bernard and coworkers [11] reported an implementation of the spin–flip (SF) variant of time-dependent density functional theory (TD-DFT) by exploring energy gaps in a variety of diradicals derived by the ring opening in cyclohexane and methyl–cyclohexane. Diradical is the most common element that forms the basis of molecular materials with a high spin state. Organic diradicals contain paramagnetic groups as spin sources and π-conjugated organic moieties as a coupler. The nature of the latter is crucial in determining the spin–spin coupling interactions between the spin sources [13]. A coupler that is extensively conjugated can give rise to a strong magnetic interaction between the spin carriers [13,14,15,16,17]. Lee et al. [15] revealed that nitronyl nitroxide radicals coupled to phenoxyl radicals via an ethylene coupler has a strong ferromagnetic interaction. It is well-known that the magnetic interactions between two radical groups can be influenced by a number of factors. As demonstrated by Ali and Datta [13], spin delocalization as well as the length and aromaticity of the couplers would affect the magnetic coupling. By twisting the carbon-carbon single bond of the coupler, Feng et al. [17] noticed that the dihedral angle could effectively modulate the spin coupling interaction by investigating the biphenyl-bridged nitroxide (NO) diradical.

In addition to the twisting effect, magnetic regulation (including magnetic switching, enhancement or weakening, etc.) can be realized through the doping effect [18], photo-induced photochromics [19,20,21], proton induction [22,23,24], temperature induction [25], redox induction [26,27,28,29], and so on [30,31,32]. Zhao et al. [21] theoretically designed a series of azobenzene-bridged diradical Janus bases, suggesting that their magnetic behaviors can convert from ferromagnetism (FM) to antiferromagnetism (AFM) based on the photochromic character of azobenzene. Moreover, Malik [23] revealed that intramolecular proton transfer can efficiently regulate the spin coupling interaction in photochromic azobenzene derivatives with an ortho-site hydroxyl as a modulator. Furthermore, non-covalent interactions, specifically hydrogen bonding (HB), were explored as a strategy to enhance the magnetic couplings of the diradicals with an acetylene-bridged phenylene coupler by Lee and co-workers [30]. In particular, Ali et al. [26] designed a redox-driven pure organic magnetic switch on the basis of the nitroxy-meta-phenylen-nitroxy diradical structure which can transform from ferromagnetic exchange coupling to antiferromagnetic exchange interaction. Our group has observed that the redox reaction can effectively modulate the magnetic properties of the diradicals in which the meta-/para-pyrazinyl act as the redox active couplers [27]. It can be concluded that, for organic diradicals, the key to magnetic regulation through redox induction is to find the coupling units with redox activity.

**Scheme 1:**
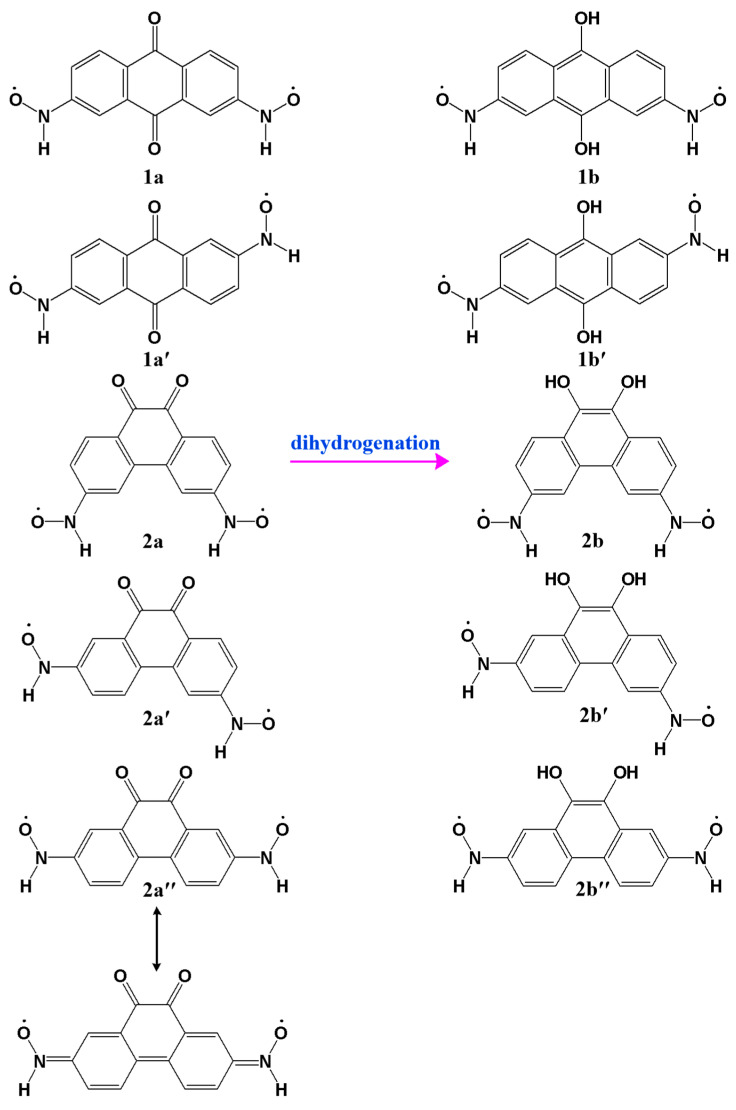

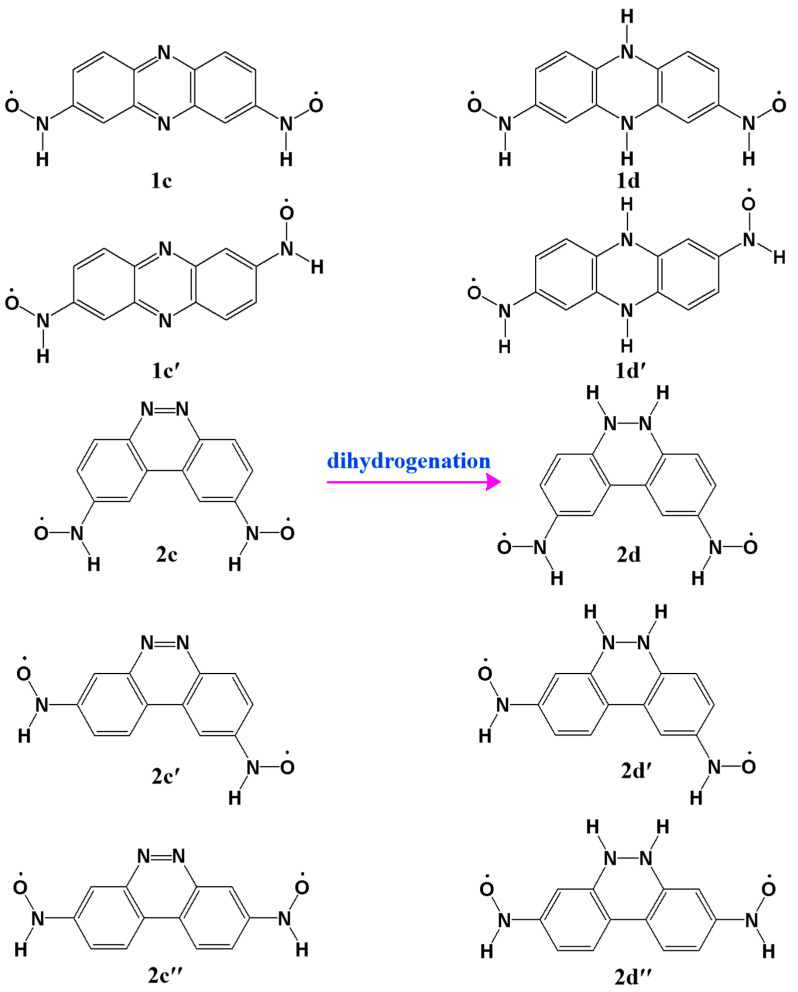
Schematic diagram of the undihydrogenated and dihydrogenated diradicals with two nitroxide (NO) radical groups. The redox-active coupling units are 9, 10-anthraquinone, 9, 10-phenaquone, 9, 10-diazanthracene and 9, 10-diazepine and their dihydrogenated counterparts. They are denoted as **1a**, **1b**, **1a′**, **1b′**, **2a**, **2b**, **2a′**, **2b′**, **2a″**, **2b″**, **1c**, **1d**, **1c′**, **1d′**, **2c**, **2d**, **2c′**, **2d′**, **2c″** and **2d″**, respectively. Moreover, the resonant structure of the diradical **2a′′** is also given.

Inspired by this, we chose 9, 10-anthraquinone and 9, 10-phenaquone as couplers with redox activity and the NO radical as spin sources. According to the different linking modes of the radical groups on the coupler, five novel diradicals are constructed, that is, **1a**, **1a′**, **2a**, **2a′** and **2a″**, as shown in Scheme 1. After dihydrogenation, **1a**, **1a′**, **2a**, **2a′** and **2a″** can convert to their reduced counterparts **1b**, **1b′**, **2b**, **2b′** and **2b″**, respectively. Besides, we also select 9, 10-diazanthracene and 9, 10-diazepines as coupling units with redox property and NO radicals as spin carriers to construct five other novel diradicals, that is, **1c**, **1c′**, **2c**, **2c′** and **2c″**, as illustrated in Scheme 1. Clearly, **1c**, **1c′**, **2c**, **2c′** and **2c″** could undergo a dihydrogenation process to convert to their reduced counterparts **1d**, **1d′**, **2d**, **2d′** and **2d″**, respectively. The calculations manifest that magnetic transitions between FM and AFM take place for four couples of designed diradicals before and after dihydrogenation (**1a** ↔ **1b**, **1a′** ↔ **1b′**, **1c** ↔ **1d** and **1c′** ↔ **1d′**). The differences in the magnetic behaviors and magnetic magnitudes of these couples of diradicals before and after dihydrogenation could be attributed to their evidently different spin-interacting pathways. Additionally, we notice that the magnetic transformation between diamagnetism (DM) and AFM occurs for one couple of designed diradicals (**2a″** ↔ **2b″**). While the signs of their magnetic exchange coupling constants *J* do not change, the magnitudes remarkably change before and after dihydrogenation for four couples of designed diradicals (**2a** ↔ **2b**, **2a′** ↔ **2b′**, **2c** ↔ **2d** and **2c″** ↔ **2d″**). Exceptionally, the magnetic magnitude of **2c′** and **2d′** is fundamentally unchanged. The connecting bond [16] linking the coupler and NO radical, spin polarization from the NO radical to coupler and favorable π-conjugated structure of the diradical are essential in the explanation of different magnetic coupling interactions of these diradicals. Specifically, shorter bonding bonds and larger spin polarization can produce stronger magnetic coupling. The diradical with an extensively π-conjugated structure is beneficial to magnetic coupling. The magnetic behaviors of FM and AFM of these diradicals obey the spin alternation rule [33,34]. Furthermore, another spin center, the tert-butyl-nitroxide radical, is also considered to verify the conclusion of the magnetic regulation induced by the redox induction for **1a**, **1b**, **1a′**, **1b′**, **2a**, **2b**, **1c**, **1d**, **1c′**, **1d′**, **2c** and **2d** (namely **1A**, **1B**, **1A′**, **1B′**, **2A**, **2B**, **1C**, **1D**, **1C′**, **1D′**, **2C** and **2D**). This work provides theoretical guidance for the rational design of redox-modulated molecular magnets and opens a new path for their applications in magnetic switches.

## 2. Results and Discussion

### 2.1. Diradical Characters and Magnetic Spin Couplings

**Figure 1:**
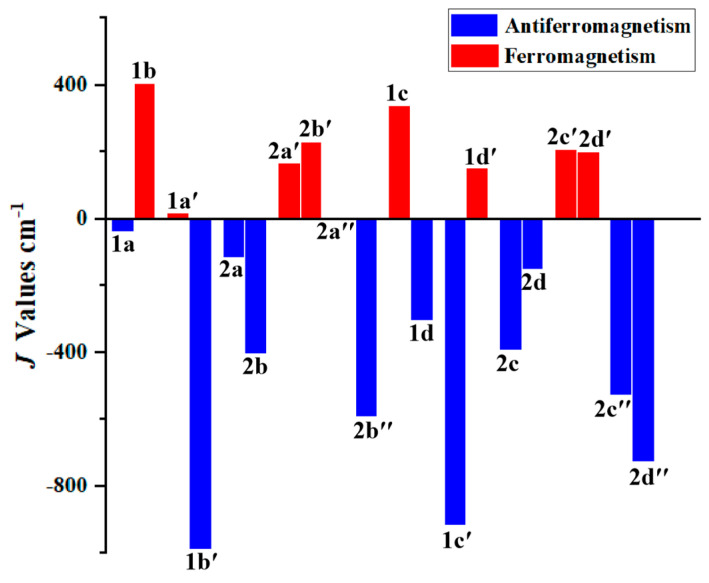
Histogram of magnetic coupling constants (*J*/cm^−1^) of **1a**, **1b**, **1a′**, **1b′**, **2a**, **2b**, **2a′**, **2b′**, **2a″**, **2b″**, **1c**, **1d**, **1c′**, **1d′**, **2c**, **2d**, **2c′**, **2d′**, **2c″** and **2d″**. The blue bars denote the diradicals with antiferromagnetism, while the red bars denote the diradicals with ferromagnetism.

We theoretically designed ten couples of NO-based molecular diradical structures (**1a** ↔ **1b**, **1a′** ↔ **1b′**, **2a** ↔ **2b**, **2a′** ↔ **2b′**, **2a″** ↔ **2b″**, **1c** ↔ **1d**, **1c′** ↔ **1d′**, **2c** ↔ **2d**, **2c′** ↔ **2d′** and **2c″** ↔ **2d″**), and discuss in detail the magnetic properties of each couple of diradicals before and after dihydrogenation, including magnetic magnitude and magnetic behavior. The detailed data, including the energies of the closed-shell singlet (CS), broken-symmetry (BS) open-shell singlet and triplet (T) states, the <S^2^> values and the magnetic exchange coupling constants *J* of all studied diradicals calculated at the B3LYP [35] function with 6-311++G(d,p) basis set are given in Table S1 in the Supplementary Materials. For the purpose of comparison, the energies of the BS and T states, <S^2^> values and *J* values estimated at the UM06-2X [36] function with 6-311++G(d,p) basis set are also presented in Table S1 (in the Supplementary Materials). It is observed that the calculation results obtained by B3LYP and M06-2X functions are relatively accurate and magnetic regulation can be achieved for each couple of diradicals before and after dihydrogenation. In the following discussion, the data used are all from the B3LYP function. From Table S1, it is found that the <S^2^> values of the diradicals of **1a**, **1b′**, **2a**, **2b**, **2b′′**, **1d**, **1c′**, **2c**, **2d**, **2c″** and **2d″** are close to 1.0 with the BS ground states, while the diradicals of **1b**, **1a′**, **2a′**, **2b′**, **1c**, **1d′**, **2c′** and **2d′** have the T ground states with the <S^2^> values close to 2.0. Undoubtedly, these indicate that the designed molecules are standard diradicals with small spin contamination and have the expected magnetic behaviors (AFM or FM). For 9, 10-anthraquinone and 9, 10-diazanthracene-bridged diradicals, a dihydrogenation process can induce the magnetic transitions between FM and AFM with the change in the position of the NO radical on the coupler. For example, **1a** and **1c′** exhibit AFM (−37.9 and −918.0 cm^−1^), while their reduced counterparts **1b** and **1d′** present FM (408.0 and 152.4 cm^−1^), as shown in Figure 1. For 9, 10-phenaquone and 9, 10-diazepine-bridged diradicals, the alternation in the position of the NO radical on the coupling unit can noticeably change the magnetic coupling magnitudes from −115.6 cm^−1^ (**2a**) to −405.2 cm^−1^ (**2b**), 166.0 cm^−1^ (**2a′**) to 230.6 cm^−1^ (**2b′**), −528.8 cm^−1^ (**2c″**) to −725.8 cm^−1^ (**2d″**) or from −394.1 cm^−1^ (**2c**) to −150.3 cm^−1^ (**2d**). Exceptionally, after dehydrogenation, the magnetic transformation from DM to AFM takes place for **2a′′** and **2b′′** (−593.2 cm^−1^), while the magnetic magnitude of **2c′** (208.9 cm^−1^) and **2d′** (201.4 cm^−1^) is basically unchanged. The histogram of *J* values of ten couples of diradicals is shown in Figure 1. The magnetic differences of ten couples of diradicals are closely related to their structural characteristics discussed below. It should be pointed out that the relevant data for the diradicals **1a**, **1b**, **1c** and **1d** were all from [29]. We also calculated the *J* values of **1a**, **1b**, **1a′**, **1b′**, **1c**, **1d**, **1c′** and **1d′** at the (U)B3LYP/6-311G(d,p) level. The relevant data are summarized in Table S2. By comparing the *J* values of **1a**, **1b**, **1a′**, **1b′**, **1c**, **1d**, **1c′** and **1d′** in Tables S1 and S2, we can find that whether the dispersion correction is added has little effect on the magnetic coupling of these diradicals. Table S3 provides the *J* values calculated for the other six couples of bis(tert-butyl)-nitroxide-based diradicals (**1A** ↔ **1B**, **1A′** ↔ **1B′**, **2A** ↔ **2B**, **1C** ↔ **1D**, **1C′** ↔ **1D′** and **2C** ↔ **2D**). The magnitudes of *J* for the tert-butyl-nitroxide-based diradicals were basically comparable to that of the corresponding nitroxide-based ones. In particular, the sign or magnitude of *J* for the tert-butyl-nitroxide-based diradicals changed after dihydrogenation, which further proved that the redox induction could achieve magnetic regulation.

### 2.2. Molecular Geometries

In general, diradicals with a good planar structure can produce a large magnetic coupling interaction [13]. Furthermore, it has been reported that the shorter the connecting bond linking the coupler and radical group, the stronger the magnetic coupling between two radical groups [16]. For **1a** and **1a′**, the geometric optimizations in Figure S1 (in the Supplementary Materials) suggest that the coupler 9, 10-anthraquinone and two NO radical groups are coplanar before and after dihydrogenation. As a result, the magnetic couplings of their reduced counterparts **1b** and **1b′** are strong, and the corresponding |*J*| values are 408.0 and 987.3 cm^−1^, respectively. Meanwhile, the magnetic interactions of **1a** (−37.9 cm^−1^) and **1a′** (17.0 cm^−1^) are weak, mainly due to the presence of the two carbonyl groups in the coupling unit blocking the spin polarization discussed below. The connecting bonds of **1a**, **1b**, **1a′** and **1b′** are 1.392, 1.387, 1.392 and 1.381 Å in sequence, and their corresponding |*J*| values are 37.9, 408.0, 17.0 and 987.3 cm^−1^, respectively, presenting a negative correlation, as shown in Table S4 (in the Supplementary Materials). For **2a**, **2a′** and **2a″**, the coupler 9, 10-phenaquone and two NO radical groups are also coplanar before and after dihydrogenation. As a consequence, the magnetic couplings of **2a**, **2b**, **2a′**, **2b′** and **2b″** are strong or moderate, and their corresponding |*J*| are 115.6, 405.2, 166.0, 230.6 and 593.2 cm^−1^, respectively. Among them, it is evident from Table S4 that the shorter connection bonds of **2b** (1.390 Å) and **2b″** (1.389 Å) correspond to stronger magnetic couplings. It is worth mentioning that **2a″** presents the shortest connecting bond (1.346 Å) of all the diradicals, implying a double bond between the linked C and linking N. Consequently, the spin coupling between two NO radical groups of **2a″** is quite intense because of a rearrangement of the C-C bonds within the coupler, which contributes to the disappearance of magnetism. That is, the spin coupling interaction is too strong, and two unpaired electrons of the radical groups would be strongly coupled and present a CS ground state so that the magnetism disappears. With respect to **1c** (*J* = 339.6 cm^−1^), **1c′** (*J* = −918.0 cm^−1^) and **2c** (*J* = −394.1 cm^−1^), the good coplanarity between two NO radicals and the coupler 9, 10-diazanthracene or 9, 10-diazepines is removed to a certain extent after dihydrogenation, so the magnetic couplings of **1d** (−302.5 cm^−1^), **1d′** (152.4 cm^−1^) and **2d** (−150.3 cm^−1^) are significantly weakened. The connecting bonds and magnetic coupling strength of **1c**, **1d**, **1c′**, **1d′**, **2c** and **2d** are negatively correlated, as displayed in Table S4. Among these six diradicals, we observe that the shortest connecting bond of **1c′** (1.383 Å) corresponds to the strongest magnetic coupling (−918.0 cm^−1^), whereas the longest connecting bond of **2d** (1.397 Å) corresponds to the weakest magnetic coupling (−150.3 cm^−1^). Moreover, the strong magnetic coupling of **2d″** (−725.8 cm^−1^) is also closely related to its short connecting bond (1.384 Å). Compared with **2c′** (*J* = 208.9 cm^−1^), after dihydrogenation, the poorer planar structure corresponds to slightly weaker magnetic coupling for **2d′** (201.4 cm^−1^). The N-O bond lengths of the studied diradicals for their ground states are shown in Table S5 for experimental reference.

### 2.3. Spin Polarization Analysis

In addition to the geometric structure characteristics, the spin polarization can better explain the magnetic differences of these ten couples of diradicals. In general, the diradical with a good π-conjugated structures is prone to spin polarization, thus effectively promoting the magnetic coupling between two radical groups [13] In other words, the spin density of the diradical with good planarity is easily delocalized from the radical group to the coupling unit, which creates a favorable condition for the magnetic coupling between two radical groups. As can be seen from Figure S2 (in the Supplementary Materials), there is almost no spin density distribution at the carbonyl carbon for **1a** and **1a′**; that is, the spin polarization is blocked at the carbonyl carbon, corresponding to weak magnetic couplings. The small |*J*| values of **1a** (37.9 cm^−1^) and **1a′** (17.0 cm^−1^) confirm this viewpoint. After dihydrogenation, the average spin density delocalization into the coupler evidently increases for **1a** and **1a′**, which corresponds to strong magnetic couplings. Specifically, 17.6% and 21.7% of spin densities are delocalized to the coupler for **1b** and **1b′**, corresponding to 408.0 and −987.3 cm^−1^, as shown in Table 1. Similarly, after dihydrogenation, the average spin density delocalization into the coupler distinctly increases for **2a** and **2c″**. The spin polarization toward the coupler is 15.9%, 16.6%, 16.0% and 19.8% for **2a**, **2b**, **2c′′** and **2d″**, which corresponds to −115.6, −405.2, −528.8 and −725.8 cm^−1^ in turn. With respect to **2b″**, the percentage of the average spin density delocalization into the coupler is large, corresponding to strong magnetic coupling. As for **1c′** and **2c**, after dihydrogenation, the spin polarization is prominently decreased from 21.0 to 16.9 and 16.3 to 15.5. Consequently, the magnetic couplings of **1d′** (152.4 cm^−1^) and **2d** (−150.3 cm^−1^) are significantly weakened compared with **1c′** (−918.0 cm^−1^) and **2c** (−394.1 cm^−1^). With regard to the remaining three couples of diradicals **2a′**, **2b′**, **1c**, **1d**, **2c′** and **2d′**, the larger spin polarization corresponds to the weaker magnetic coupling, and the smaller spin polarization corresponds to the stronger magnetic coupling, which indicates that the spin polarization is not the only factor affecting the magnetic coupling for these diradicals. The average spin density delocalization into the coupler of ten couples of diradicals and their corresponding *J* values are listed in Table 1. The Mulliken atomic spin density distributions of ten couples of diradicals are depicted in Figure S2.

### 2.4. Spin Coupling Pathways and Spin Alternation Rule Analysis

**Figure 2:**
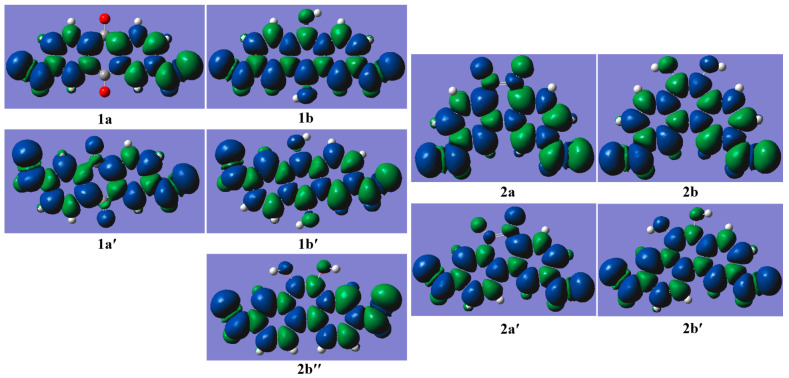

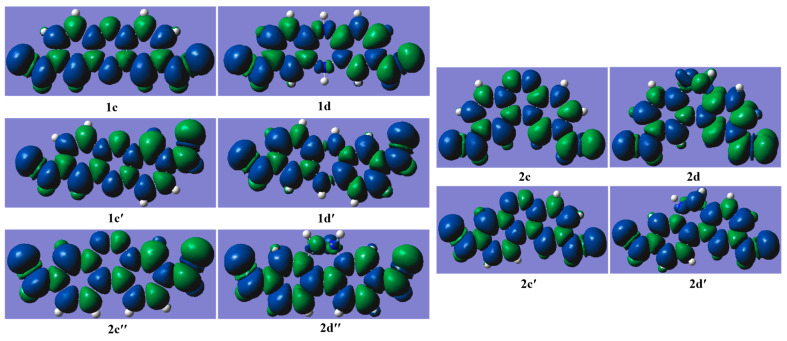
Spin density distributions (isovalue = 0.0004) of the ground states of **1a**, **1b**, **1a′**, **1b′**, **2a**, **2b**, **2a′**, **2b′**, **2b″**, **1c**, **1d**, **1c′**, **1d′**, **2c**, **2d**, **2c′**, **2d′**, **2c″** and **2d″**. The blue colour indicates spin up and the green colour indicates spin down.

**Figure 3:**
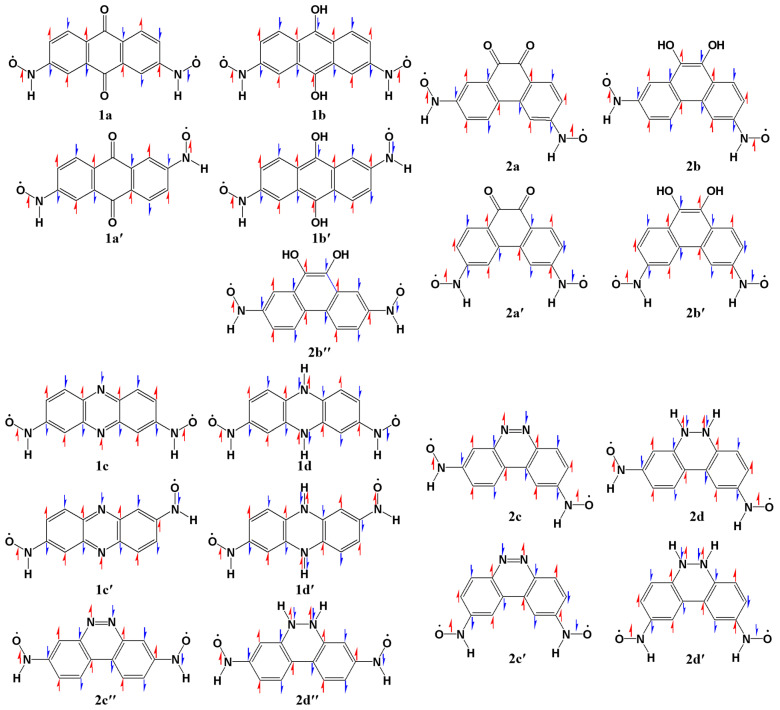
Scheme of spin alteration for **1a**, **1b**, **1a′**, **1b′**, **2a**, **2b**, **2a′**, **2b′**, **2b″**, **1c**, **1d**, **1c′**, **1d′**, **2c**, **2d**, **2c′**, **2d′**, **2c″** and **2d″**. The red arrow indicates spin up and the blue arrow indicates spin down.

The spin density distributions of the ground states of ten couples of diradicals are shown in Figure 2. It can be seen that the spin coupling pathways of each couple of diradicals were visibly different before and after dihydrogenation. For 9, 10-anthraquinone-bridged diradicals **1a** and **1a′**, the spin coupling pathway was clearly blocked at the carbonyl carbon, which hindered the spin transmission through the coupler. Consequently, the magnetic couplings of **1a** and **1a′** were greatly weakened, corresponding to −37.9 and 17.0 cm^−1^. After dihydrogenation, the spin transport from the radical group to the coupling unit was unimpeded, thus effectually promoting the magnetic coupling. The strong magnetic coupling of **1b** (408.0 cm^−1^) and **1b′** (−987.3 cm^−1^) was in good conformity with the above opinion. Interestingly, we also observe that the magnetic conversion between FM and AFM couplings occurs for **1a** and **1b,** as well as **1a′** and **1b′**. The adjacent atomic center exhibits opposite spins for **1b** and **1b′**, obeying the spin alternation rule. The rule states that the sign of *J* depends on the parity of the number of bonds in the coupling pathway through the coupler. When the number is odd, *J* is negative. On the contrary, if the number of bonds is even, *J* is positive. According to this spin alternation rule, **1b** presents AFM coupling, while AFM coupling occurs for **1b′**. This is in good agreement with the calculated results. As for 9, 10-phenaquone-bridged diradicals **2a**, **2a′** and **2a″**, the spin polarization was not blocked at the carbonyl carbon, so only the magnetic magnitudes of these diradicals changed after dihydrogenation, and their magnetic behaviors did not change by means of the spin alternation rule. Abnormally, **2a″** exhibited DM behaviors due to the strong interaction between two radical groups, as already mentioned above. It can be concluded that, after dihydrogenation, magnetic conversion can be induced for 9, 10-anthraquinone-bridged diradicals, while only the magnetic magnitude can be regulated for 9, 10-phenaquone-bridged diradicals. On the basis of the spin alternation rule, **1c** would exhibit FM, while **1c′** would exhibit AFM. After dihydrogenation, we found that, through the coupling pathway, each of the two nitrogen atoms in the coupler could provide two π-electrons, equivalent to a chemical bond. As a result, the numbers of bonds through the coupling pathway of **1d** was odd, presenting AFM coupling. Meanwhile, the numbers of bonds through the coupling pathway of **1d′** were even and an FM coupling arose. With respect to 9, 10-diazepines-bridged diradicals **2c**, **2c′** and **2c″**, there were three main coupling pathways though the coupler and the parity of the number of bonds through the coupling pathway remained unchanged after dihydrogenation. It is worth mentioning that in one of the coupling pathways, two nitrogen atoms of the reduced counterparts of **2c**, **2c′** and **2c″** could each provide two nitrogen atoms, equivalent to two chemical bonds. Thus, for **2c**, **2c′** and **2c′′**, after dihydrogenation, the parity of the number of bonds through three coupling pathways remained unchanged. As a consequence, the magnetic magnitudes of **2c**, **2c′** and **2c″** changed after dihydrogenation, while their magnetic behaviors remained the same. We can draw a conclusion that after dihydrogenation, magnetic transformation can occur for 9, 10-diazanthracene-bridged diradicals, while only the magnetic magnitude can be modulated for 9, 10-diazepines-bridged diradicals. The spin alternation schemes of ten couples of diradicals are shown in Figure 3. Furthermore, the energetically unfavorable spin states for all the studied diradicals are shown in Figure S3, where the spin polarizations are blocked through the couplers.

## 3. Computational Details

All the molecular geometric optimizations and frequency analyses, as well as energy calculations of the closed-shell (CS) singlet, broken-symmetry (BS) open-shell singlet and triplet (T) states are performed at the (U)B3LYP/6-311++G(d,p) level of theory. Additionally, a more modern M06-2X function with a 6-311++G(d,p) basis set is employed to verify the accuracy of some computational results, which has certain advantages in dealing with weak interactions. Furthermore, to discuss the effects of not adding the dispersion correction in the DFT calculations, we also calculated the magnetic couplings of 9, 10-anthraquinone and 9, 10-diazanthracene-bridged nitroxide-based diradicals at the (U)B3LYP/6-311G(d,p) level. Moreover, to demonstrate the redox-driven magnetic regulation, the magnetic couplings of six couples of bis(tert-butyl)-nitroxide-based diradicals are also considered at the (U)B3LYP/6-311++G(d,p) level. In general, very small spin contamination occurs for the high-spin triplet state. In contrast, the BS state is often found as spin-contaminated. Therefore, spin-projected methods were applied to eliminate the effect of the spin contamination from the energy of the BS state. The equation of *J* is the result obtained from these methods: *J* = (E_BS_ − E_T_)/(<S^2^>_T_ − <S^2^>_BS_), where E_BS_ and E_T_ refer to the energies of the BS open-shell singlet and triplet state, while <S^2^>_BS_ and <S^2^>_T_ denote the average spin square values of the two states, respectively. This equation was proposed by Yamaguchi and co-workers and regarded as the most appropriate evaluation of the *J* value [37,38]. All of these DFT calculations were carried out by adopting the Gaussian 09 suite of the program [39].

## 4. Conclusions

In summary, we theoretically design ten couples of NO-based diradicals with 9, 10-anthraquinone, 9, 10-phenaquone, 9, 10-diazanthracene and 9, 10-diazepines as the coupler, and discuss in detail the redox-induced magnetic regulation of each couple of diradicals. For 9, 10-anthraquinone and 9, 10-diazanthracene-bridged diradicals, after dihydrogenation, magnetic switching between FM and AFM coupling is observed (**1a** ↔ **1b**, **1a′** ↔ **1b′**, **1c** ↔ **1d** and **1c′** ↔ **1d′**). The differences in the magnetic behaviors and magnetic magnitudes of these four couples of diradicals before and after dihydrogenation are closely related to their different spin-interacting pathways. For 9, 10-anthraquinone-bridged diradicals **1a** and **1a′**, the presence of the carbonyl group in the coupling path blocks the spin transport, causing a magnetic transition and leading to weak magnetic couplings. For 9, 10-diazanthracene-bridged diradicals **1c** and **1c′**, after dihydrogenation, the planarity of **1d** and **1d′** becomes worse and their corresponding magnetic couplings are weakened. As for 9, 10-phenaquone and 9, 10-diazepines-bridged diradicals, after dihydrogenation we notice that the magnetic enhancement takes place for three couples of diradicals (**2a** ↔ **2b**, **2a′** ↔ **2b′** and **2c″** ↔ **2d″**), while the magnetic weakening occurs for one couple of diradicals (**2c** ↔ **2d**). Exceptionally, after dihydrogenation, magnetic transformation from DM to AFM occurs for **2a″** and **2b″**, while the magnetic magnitude of **2c′** and **2d′** is basically unchanged. The geometric structure and spin polarization are helpful in explaining the different magnetic magnitudes of these diradicals. The good planar structure, short connecting bond and large spin polarization can greatly promote the magnetic coupling. Among ten couples of diradicals, except for **2a″**, for **1d′**, its good planar structure, the shortest connecting bond (1.381 Å), and the largest spin polarization (21.7%) corresponds to the strongest magnetic coupling (−987.3 cm^−1^). The magnetic behaviors of all these studied diradicals can be qualitatively predicted by means of the spin alternation rule. This work may broaden the landscape for the rational design of magnetic molecular switches.

## Figures and Tables

**Table 1:** Average spin density delocalization into coupler (%) and corresponding intramolecular magnetic coupling constants (*J*, in cm^−1^) for all studied nitroxide-based diradicals.

Species	Average Spin Density Delocalization into Coupler (%)	*J*	Species	Average Spin Density Delocalization into Coupler (%)	*J*
**1a**	15.1	−37.9	**1b**	17.6	408.0
**1a′**	15.0	17.0	**1b′**	21.7	−987.3
**2a**	15.9	−115.6	**2b**	16.6	−405.2
**2a′**	15.4	166.0	**2b′**	14.9	230.6
**2a″**	—	—	**2b″**	16.9	−593.2
**1c**	16.9	339.6	**1d**	17.8	−302.5
**1c′**	21.0	−918.0	**1d′**	16.9	152.4
**2c**	16.3	−394.1	**2d**	15.5	−150.3
**2c′**	14.5	208.9	**2d′**	15.8	201.4
**2c″**	16.0	−528.8	**2d″**	19.8	−725.8

## Data Availability

The data that support the findings of this study are available from the corresponding author upon reasonable request.

## Supplementary Materials

The following supporting information can be downloaded at: https://www.mdpi.com/article/10.3390/molecules30030576/s1: Relevant Data of Describing Ground States for All Designed Diradicals, Their Diradical Character and Magnetic Behaviors (Tables S1, S2 and S3); Connecting Bond Linking Coupler and Nitroxide Group for All Diradicals with Ground States and Corresponding *J* Values, Bond Length of N-O group for All Diradicals with Ground States (Tables S4 and S5); Optimized Molecular Geometries for Ground States of All Diradicals (Figure S1); Distributions of Mulliken Atomic Spin Density of All Diradicals (Figure S2); Spin Density Maps of Energetically Unfavorable Spin States for All Diradicals (Figure S3).
