# Supplementary material for: Redox-Driven Magnetic Regulation in a Series of Couplers in Bridged Nitroxide Diradicals
Source: Molecules. 2025 Jan 27;30(3):576. doi: 10.3390/molecules30030576 (PMC11819652; doi:10.3390/molecules30030576)
Supplement: Supplementary file 1 [file molecules-30-00576-s001.zip › molecules-3420209-supplementary.pdf]

Supporting Information

Contents

1. Relevant Data of Describing Ground States for All Designed Diradicals, Their Diradical Character and Magnetic Behaviors

2. Connecting Bond Linking Coupler and Nitroxide Group for All Diradicals with Ground States and Corresponding *J* Values, Bond Length of N-O group for All Diradicals with Ground States

3. Optimized Molecular Geometries for Ground States of All Diradicals

4. Distributions of Mulliken Atomic Spin Density of All Diradicals

5. Spin Density Maps of Energetically Unfavorable Spin States for All Diradicals

1. Relevant Data of Describing Ground States for All Designed Diradicals, Their Diradical Character and Magnetic Behaviors

Table S1. (U)B3LYP/6-311++G(d,p)* and (U)M06-2X/6-311++G(d,p)** levels estimated energies (in a.u.) of the closed-shell (CS) singlet, broken-symmetry (BS) open-shell singlet and triplet (T) state, corresponding <S^2> values as well as intramolecular magnetic coupling constants (*J*, in cm^{-1}) for all the studied diradicals.

Species	$E_{(CS)}$*	$E_{(BS)}$ (<S^2>)*	$E_{(BS)}$ (<S^2>)**	*J**	*J***	$E_{(T)}$ (<S^2>)*	$E_{(T)}$ (<S^2>)**
**1a**	-948.8084098	-948.8452382(1.026)	-948.4710141(1.030)	-37.9	-4.7	-948.8450641(2.034)	-948.4709925(2.034)
**1b**	-950.0009064	-950.0400077(1.022)	-949.6639157(1.026)	408.0	276.4	-950.0419894(2.087)	-949.6652353(2.073)
**1a′**	-948.7988940	-948.8438107(1.033)	-948.4696853(1.033)	17.0	0.99	-948.8438880(2.033)	-948.4696808(2.032)
**1b′**	-950.0286137	-950.0432985(1.007)	-949.6653563(1.061)	-987.3	-643.9	-950.0387242(2.023)	-949.6625288(2.024)
**2a**	-948.7888318	-948.8253725(1.039)	-948.4513641(1.039)	-115.6	-62.1	-948.8248502(2.030)	-948.4510836(2.030)
**2b**	-950.0197265	-950.0425892(1.020)	-949.6668270(1.036)	-405.2	-215.7	-950.0407396(2.021)	-949.6658560(2.023)
**2a′**	-948.7803931	-948.8227178(1.025)	-948.4491616(1.027)	166.0	106.2	-948.8234899(2.045)	-948.4496522(2.040)
**2b′**	-949.9980280	-950.0420629(1.020)	-949.6672319(1.022)	230.6	146.3	-950.0431406(2.045)	-949.6679103(2.039)
**2a″**	-948.8195863	—	—	—	—	-948.8026782(2.020)	-948.4464691(2.021)
**2b″**	-950.0241736	-950.0452053(0.997)	-949.6695335(1.027)	-593.2	-374.8	-950.0424436(2.018)	-949.6678365(2.020)
**1c**	-831.5986800	-831.6375954(1.023)	-831.3019635(1.027)	339.6	234.0	-831.6392154(2.069)	-831.3030659(2.060)
**1d**	-832.8271046	-832.8500366(1.015)	-832.5121497(1.033)	-302.5	-102.3	-832.8486393(2.028)	-832.5116849(2.029)
**1c′**	-831.6240969	-831.6398810(0.994)	-831.3029673(1.047)	-918.0	-615.0	-831.6355776(2.022)	-831.3002273(2.024)
**1d′**	-832.8132673	-832.8507064(1.028)	-832.5133578(1.030)	152.4	62.2	-832.8514126(2.044)	-832.5136433(2.037)
**2c**	-831.5849087	-831.6090777(1.021)	-831.2744915(1.035)	-394.1	-224.4	-831.6072823(2.020)	-831.2734815(2.022)
**2d**	-832.7774416	-832.8082243(1.035)	-832.4708275(1.036)	-150.3	-67.6	-832.8075443(2.027)	-832.4705218(2.027)
**2c′**	-831.5615289	-831.6059427(1.019)	-831.2721371(1.022)	208.9	138.6	-831.6069184(2.043)	-831.2727792(2.038)
**2d′**	-832.7728328	-832.8096808(1.023)	-832.4727371(1.025)	201.4	120.3	-832.8106202(2.046)	-832.4732935(2.039)
**2c″**	-831.5825836	-831.6060260(1.002)	-831.2715435(1.026)	-528.8	-345.7	-831.6035736(2.019)	-831.2699763(2.020)
**2d″**	-832.7991640	-832.8144333(0.982)	-832.4762158(1.030)	-725.8	-400.6	-832.8109946(2.021)	-832.4744034(2.022)

Table S2. (U)B3LYP/6-311G(d,p) level estimated energies (in a.u.) of the broken-symmetry (BS) open-shell singlet and triplet (T) state, corresponding <S^2> values, intramolecular magnetic coupling constants (*J*, in cm^{-1}) for four couples of diradicals.

Species	$E_{(BS)}$ (<S^2>)	$E_{(T)}$ (<S^2>)	*J*
**1a**	-948.8251058(1.025)	-948.8249510(2.033)	-33.7
**1b**	-950.0163935(1.021)	-950.0182933(2.083)	392.3
**1a′**	-948.8237728(1.032)	-948.8238502(2.031)	17.0
**1b′**	-950.0191706(1.015)	-950.0148714(2.021)	-937.1
**1c**	-831.6187312(1.021)	-831.6202786(2.066)	324.7
**1d**	-832.8296476(1.015)	-832.8281032(2.026)	-335.0
**1c′**	-831.6206765(0.998)	-831.6164806(2.020)	-900.3
**1d′**	-832.8302418(1.026)	-832.8310462(2.045)	173.1

Table S3. (U)B3LYP/6-311++G(d,p) level estimated energies (in a.u.) of the broken-symmetry (BS) open-shell singlet and triplet (T) state, corresponding <S^2> values, intramolecular magnetic coupling constants (J, in cm^{-1}) for six couples of bis(tert-butyl)-nitroxide-based diradicals.

**Species**	$E_{(BS)}$ (<S^2>)	$E_{(T)}$ (<S^2>)	J
**1A**	-1263.4271834(1.022)	-1263.4269754(2.030)	-45.2
**1B**	-1264.6185796(1.021)	-1264.6201755(2.074)	332.3
**1A′**	-1263.4248106(1.029)	-1263.4249151(2.029)	22.9
**1B′**	-1264.6207399(1.014)	-1264.6170930(2.019)	-795.7
**2A**	-1263.4045608(1.033)	-1263.4040692(2.026)	-108.6
**2B**	-1264.6205264(1.022)	-1264.6191915(2.018)	-293.9
**1C**	-1146.2180983(1.019)	-1146.2194055(2.059)	275.6
**1D**	-1147.4265221(1.019)	-1147.4255500(2.024)	-212.1
**1C′**	-1146.2183721(0.997)	-1146.2147474(2.019)	-777.7
**1D′**	-1147.4278786(1.025)	-1147.4283689(2.038)	106.1
**2C**	-1146.1879862(1.019)	-1146.1866042(2.017)	-303.7
**2D**	-1147.3896308(1.024)	-1147.3894838(2.019)	-32.4

2. Connecting Bond Linking Coupler and Nitroxide Group for All Diradicals with Ground States and Corresponding J Values, Bond Length of N-O group for All Diradicals with Ground States

Table S4. The average connecting bond length (Å) linking the coupler and nitroxide group of the studied diradicals with their ground states at the (U)B3LYP/6-311++G(d,p) level, and their corresponding J values are also listed.

**Species**	**Average Connecting Bond length (C-N)**	J	**Species**	**Average Connecting Bond length (C-N)**	J
**1a**	1.392	-37.9	**1b**	1.387	408.0
**1a′**	1.392	17.0	**1b′**	1.381	-987.3
**2a**	1.391	-115.6	**2b**	1.390	-405.2
**2a′**	1.391	166.0	**2b′**	1.392	230.6
**2a″**	1.346	—	**2b″**	1.389	-593.2
**1c**	1.388	339.6	**1d**	1.389	-302.5
**1c′**	1.383	-918.0	**1d′**	1.390	152.4
**2c**	1.390	-394.1	**2d**	1.397	-150.3
**2c′**	1.393	208.9	**2d′**	1.392	201.4
**2c″**	1.391	-528.8	**2d″**	1.384	-725.8

Table S5. The average N-O bond length (Å) of the studied diradicals with their ground states at the (U)B3LYP/6-311++G(d,p) level.

Species	Average Bond length (N-O)	Species	Average Bond length (N-O)
**1a**	1.270	**1b**	1.274
**1a′**	1.269	**1b′**	1.273
**2a**	1.269	**2b**	1.274
**2a′**	1.270	**2b′**	1.273
**2a″**	1.262	**2b″**	1.272
**1c**	1.271	**1d**	1.276
**1c′**	1.269	**1d′**	1.276
**2c**	1.270	**2d**	1.274
**2c′**	1.271	**2d′**	1.275
**2c″**	1.271	**2d″**	1.274

3. Optimized Molecular Geometries for Ground States of All Diradicals

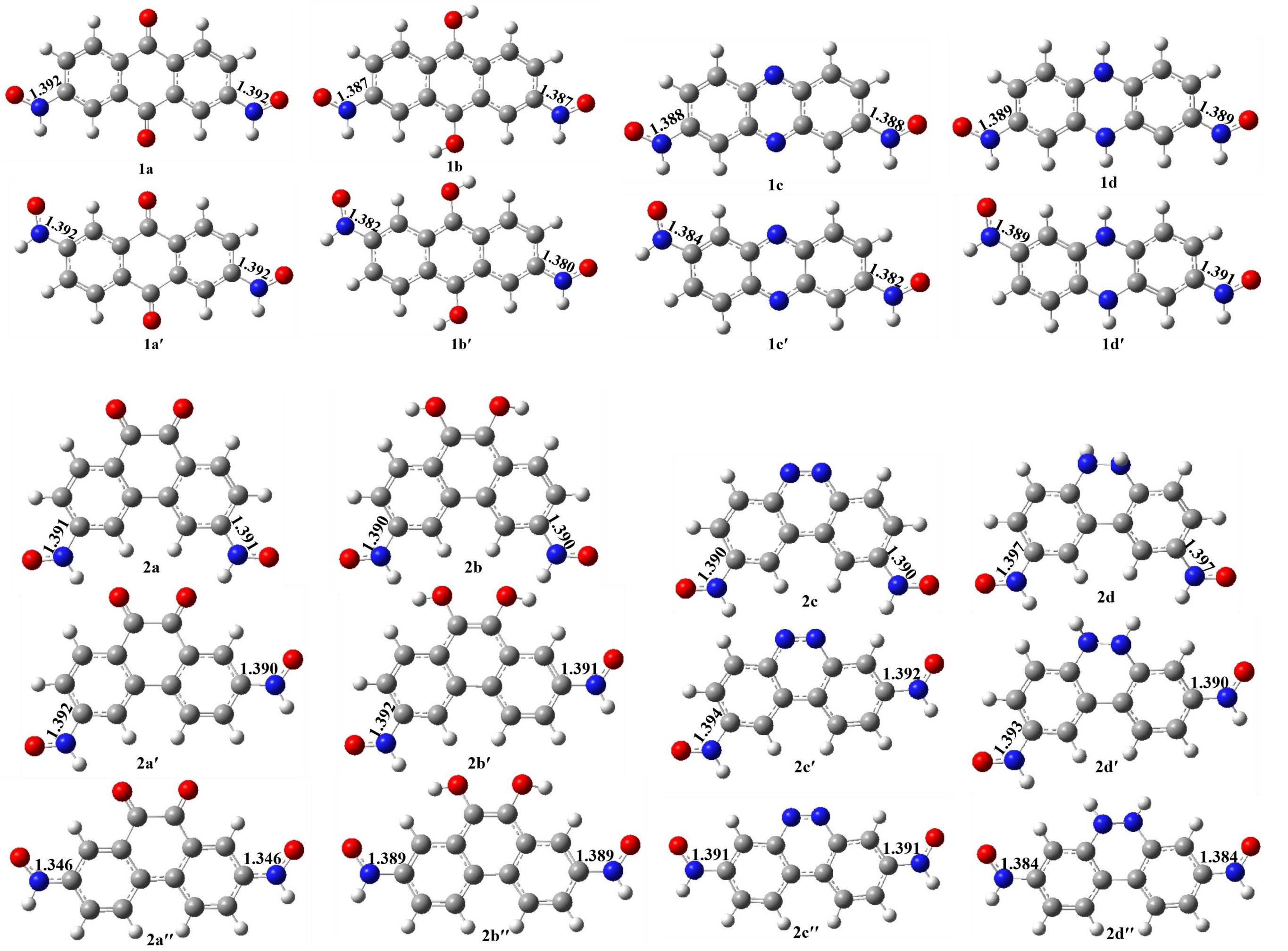

Figure S1. Optimized geometries for the ground states of all diraidcals at the (U)B3LYP/6-311++G (d,p) level, including two linking C-N bonds.

4. Distributions of Mulliken Atomic Spin Density of All Diradicals

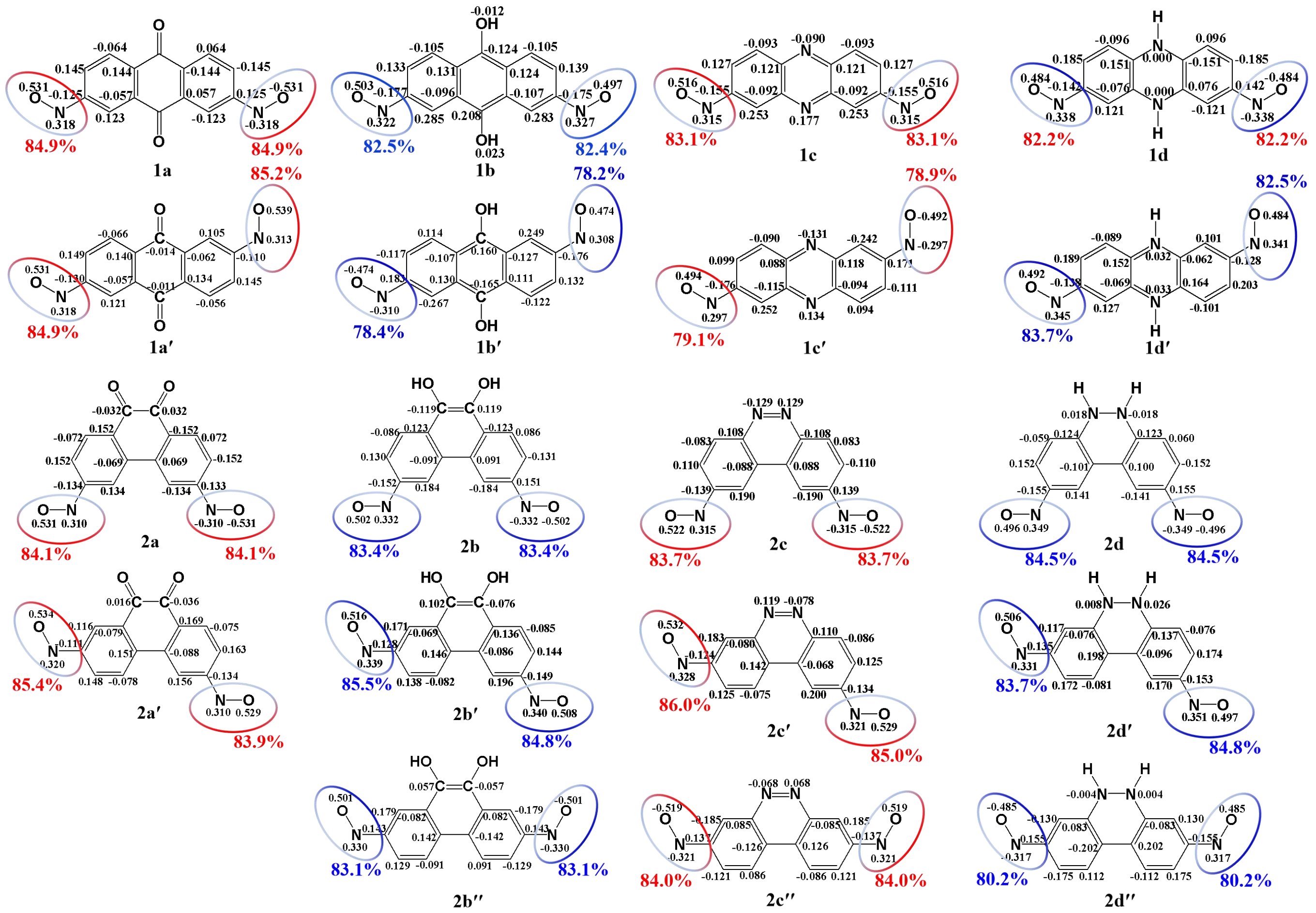

Figure S2. Comparison of spin polarization and the Mulliken atomic spin density distributions of all diradicals before and after dihydrogenation.

5. Spin Density Maps of Energetically Unfavorable Spin States for All Diradicals

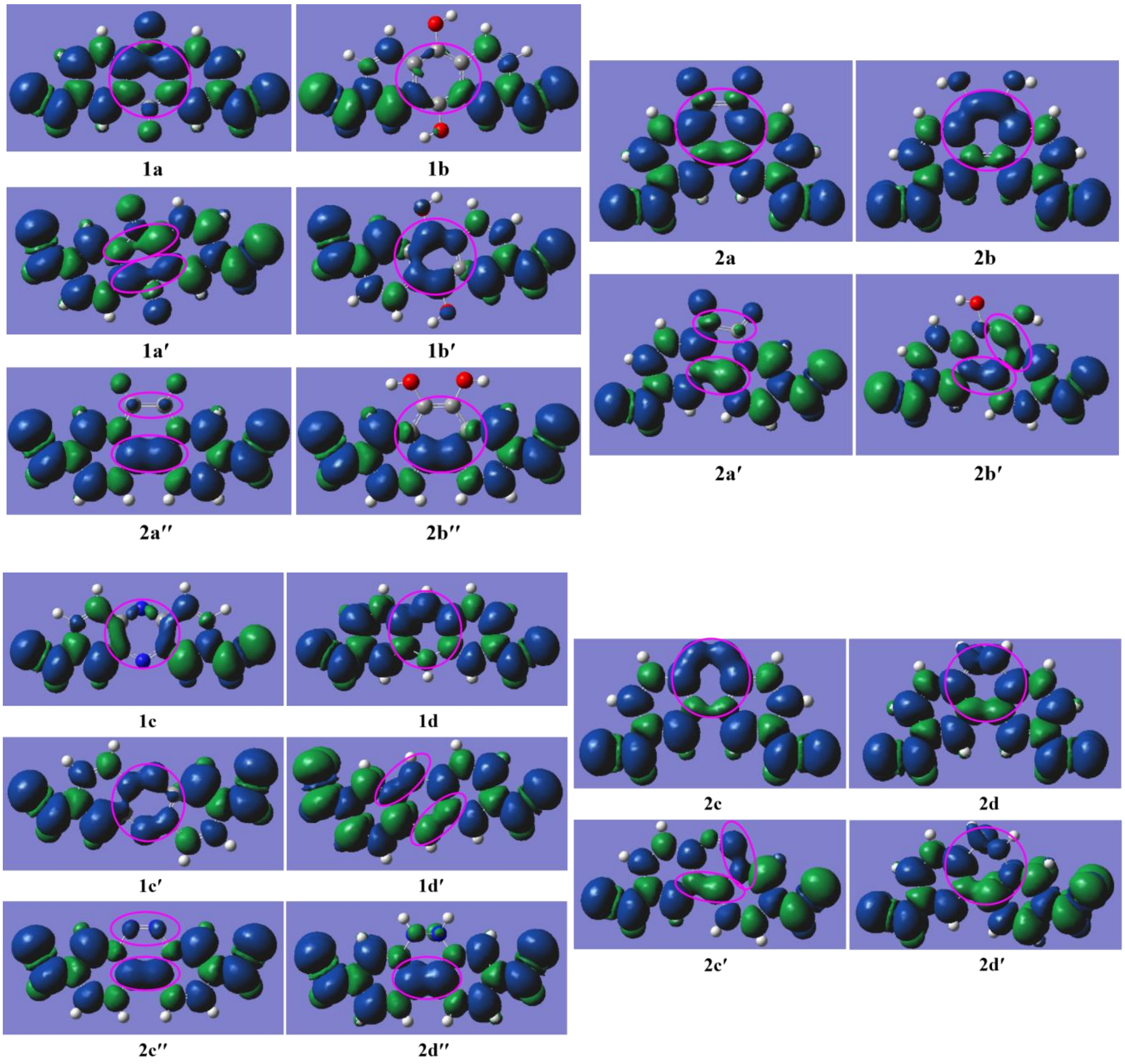

Figure S3. Spin density plots (isovalue = 0.0004) of energetically unfavorable spin states (not the ground states) for all diradicals. It can be seen that the spin polarizations through the couplers are all blocked marked with magenta circles.
